# [2-(Dimethyl­amino)­ethanol-κ^2^ *N*,*O*][2-(dimethyl­amino)­ethano­lato-κ^2^ *N*,*O*]iodidocopper(II)

**DOI:** 10.1107/S1600536812010215

**Published:** 2012-03-14

**Authors:** Elena A. Buvaylo, Volodymyr N. Kokozay, Olga Yu. Vassilyeva, Brian W. Skelton

**Affiliations:** a Department of Inorganic Chemistry, Taras Shevchenko National University of Kyiv, Volodymyrska str. 64, Kyiv 01033, Ukraine; b Centre for Microscopy, Characterisation and Analysis, University of Western Australia, 35 Stirling Highway, Crawley, WA 6009, Australia

## Abstract

The title compound, [Cu(C_4_H_10_NO)I(C_4_H_11_NO)], was obtained unintentionally as the product of an attempted synthesis of a Cu/Zn mixed-metal complex using zerovalent copper, zinc(II) oxide and ammonium iodide in pure 2-(dimethyl­amino)­ethanol, in air. The mol­ecular complex has no crystallographically imposed symmetry. The coordination geometry around the metal atom is distorted square-pyramidal. The equatorial coordination around copper involves donor atoms of the bidentate chelating 2-(dimethyl­amino)­ethanol ligand and the 2-(dimethyl­amino)­ethano­late group, which are mutually *trans* to each other, with four approximately equal short Cu—O/N bond distances. The axial Cu—I bond is substanti­ally elongated. Inter­molecular hydrogen-bonding inter­actions involving the –OH group of the neutral 2-(dimethyl­amino)­ethanol ligand to the O atom of the monodeprotonated 2-(dimethyl­amino)­ethano­late group of the mol­ecule related by the *n*-glide plane, as indicated by the O⋯O distance of 2.482 (12) Å, form chains of mol­ecules propagating along [101].

## Related literature

For background to the synthesis, see: Vinogradova *et al.* (2002 ▶). Buvaylo *et al.* (2009 ▶, 2011 ▶). Elongation of the axial Cu—I bond is common in this kind of compound, see: Wells (1986 ▶).
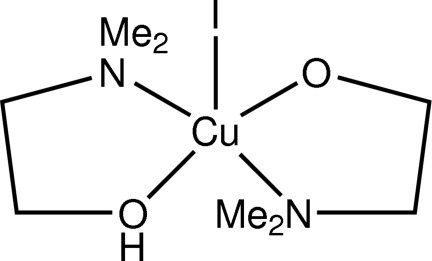


## Experimental

### 

#### Crystal data

[Cu(C_4_H_10_NO)I(C_4_H_11_NO)]
*M* *_r_* = 367.71
Monoclinic, 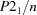

*a* = 8.690 (1) Å
*b* = 15.241 (1) Å
*c* = 11.116 (1) Å
β = 106.847 (10)°
*V* = 1409.1 (2) Å^3^
*Z* = 4
Mo *K*α radiation
μ = 3.72 mm^−1^
*T* = 296 K
0.32 × 0.3 × 0.2 mm

#### Data collection

Rigaku AFC-6S diffractometer
Absorption correction: ψ scan (North *et al.*, 1968 ▶) *T*_min_ = 0.333, *T*_max_ = 0.47
2642 measured reflections
2471 independent reflections
1003 reflections with *I* > 2σ(*I*)
*R*_int_ = 0.089
3 standard reflections every 150 reflections intensity decay: none

#### Refinement

*R*[*F* ^2^ > 2σ(*F* ^2^)] = 0.058
*wR*(*F* ^2^) = 0.204
*S* = 1.00
2471 reflections
133 parameters
H-atom parameters constrained
Δρ_max_ = 1.80 e Å^−3^
Δρ_min_ = −0.83 e Å^−3^

### 

Data collection: *AFC6S Diffractometer Control Software* (Molecular Structure Corporation, 1998) ▶; cell refinement: *AFC6S Diffractometer Control Software*; data reduction: *TEXSAN* (Molec­ular Structure Corporation & Rigaku, 2000 ▶); program(s) used to solve structure: *SHELXS97* (Sheldrick, 2008 ▶); program(s) used to refine structure: *SHELXL97* (Sheldrick, 2008 ▶); molecular graphics: Johnson (1976 ▶); software used to prepare material for publication: *WinGX* (Farrugia, 1999 ▶).

## Supplementary Material

Crystal structure: contains datablock(s) I, global. DOI: 10.1107/S1600536812010215/ds2178sup1.cif

Structure factors: contains datablock(s) I. DOI: 10.1107/S1600536812010215/ds2178Isup2.hkl

Additional supplementary materials:  crystallographic information; 3D view; checkCIF report

## Figures and Tables

**Table 1:** Selected bond lengths (Å)

I1—Cu1	2.928 (2)
Cu1—O1	2.030 (9)
Cu1—N2	2.058 (11)
Cu1—N1	2.059 (10)
Cu1—O2	2.010 (8)

**Table 2:** Hydrogen-bond geometry (Å, °)

*D*—H⋯*A*	*D*—H	H⋯*A*	*D*⋯*A*	*D*—H⋯*A*
O2—H2*O*⋯O1^i^	0.82	1.68	2.482 (12)	167

Symmetry code: (i) 
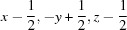
.

**Fig. 1.**
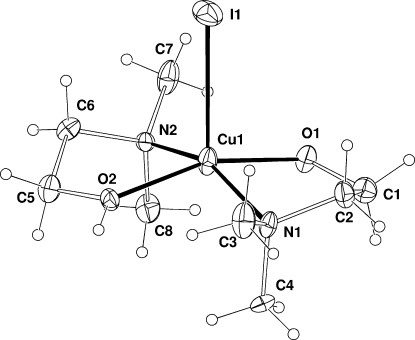
Molecular structure of the complex with the numbering scheme (the non-hydrogen atoms shown as 20% thermal ellipsoids).

**Fig. 2.**
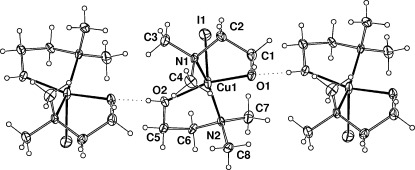
Hydrogen-bonding interactions between Cu(Me2Ea)(HMe2Ea)I molecules within a polymeric chain.

## supplementary crystallographic information

### Comment

In our previous study metal powders of zinc and copper were found to react with ammonium iodide and 2-(dimethylamino)ethanol (HMe_2_Ea) in methanol, in air, affording the new heterotrinuclear complex [Cu_2_Zn(NH_3_)I_3_(Me_2_Ea)_3_] (Vinogradova *et al.*, 2002). Reactions employing elemental copper and aminoalcohol allow *in situ* formation of the metal aminoalkoxo species - key building blocks that can subsequently self-assemble with other metal centres present in the reaction vessel (Buvaylo *et al.*, 2009; Buvaylo *et al.*, 2011). The title compound was isolated from the solution obtained by reacting copper powder and zinc oxide with ammonium iodide in pure 2-(dimethylamino)ethanol. It can be considered as an intermediate, a building block that failed self-organization with another metal species produced in the reaction medium. To the best of our knowledge the title compound has not been structurally characterized.

The molecular complex has no crystallographically imposed symmetry (Fig. 1). The coordination geometry around the metal atom is distorted square pyramidal. The equatorial coordination around Cu(1) involves donor atoms of bidentate chelating 2-(dimethylamino)ethanol and 2-(dimethylamino)ethanolato group, which are mutually *trans* to each other, with four approximately equal short distances. The axial Cu(1)–I(1) bond is substantially elongated [2.928 (2) Å], and it is common for this kind of compounds (Wells, 1986).

Intermolecular hydrogen-bonding interactions from the OH group of neutral 2-(dimethylamino)ethanol ligand to O atom of monodeprotonated 2-(dimethylamino)ethanolato group of the molecule related by the *n* glide plane [H2O···O1{*x* - 1/2, -*y* + 1/2, *z* - 1/2} = 1.68 Å; O2···O1{*x* - 1/2, -*y* + 1/2, *z* - 1/2} = 2.482 (12) Å and O2—H2O···O1{*x* - 1/2, -*y* + 1/2, *z* - 1/2} = 167.3°] form chains of Cu(Me_2_Ea)(HMe_2_Ea)I molecules propagated along the 101 direction (Fig. 2). Cu···Cu separations in the crystal are > 6.6 Å.

### Experimental

Copper powder (0.16 g, 2.5 mmol), ZnO (0.20 g, 2.5 mmol), NH_4_I (1.45 g, 10 mmol), HMe_2_Ea (15 ml) were heated to 323–333 K and magnetically stirred until total dissolution of the copper and ZnO was observed (95 min). The resulting blue solution was filtered and allowed to stand at room temperature. Green-blue microcrystals of the title compound were formed after one day. They were collected by filter-suction, washed with dry Pr^i^OH and finally dried *in vacuo* (yield: 0.23 g).

### Refinement

The non-hydrogen atoms were refined anisotropically. The hydrogen atom on O2 atom was located and its position constrained with the isotropic displacement parameter allowed to refine. Other hydrogen atoms were placed at idealized positions (C–H = 0.95 Å, *U*_iso_H = 1.20*U*_eq_ C for CH_2_, 1.5*U*_eq_ C for CH_3_) and not refined.

### Crystal data

**Table:**

[Cu(C_4_H_10_NO)I(C_4_H_11_NO)]	*F*(000) = 724
*M**_r_* = 367.71	*D*_x_ = 1.733 Mg m^−^^3^
Monoclinic, *P*2_1_/*n*	Mo *K*α radiation, λ = 0.71069 Å
Hall symbol: -p 2yn	Cell parameters from 6 reflections
*a* = 8.690 (1) Å	θ = 10.9–11.9°
*b* = 15.241 (1) Å	µ = 3.72 mm^−^^1^
*c* = 11.116 (1) Å	*T* = 296 K
β = 106.847 (10)°	Rod, blue-green
*V* = 1409.1 (2) Å^3^	0.32 × 0.3 × 0.2 mm
*Z* = 4	

### Data collection

**Table:**

Rigaku AFC-6S diffractometer	1003 reflections with *I* > 2σ(*I*)
Radiation source: fine-focus sealed tube	*R*_int_ = 0.089
Graphite monochromator	θ_max_ = 25°, θ_min_ = 2.3°
2θ–ω scans	*h* = 0→10
Absorption correction: ψ scan (North *et al.*, 1968)	*k* = 0→18
*T*_min_ = 0.333, *T*_max_ = 0.47	*l* = −13→12
2642 measured reflections	3 standard reflections every 150 reflections
2471 independent reflections	intensity decay: none

### Refinement

**Table:**

Refinement on *F*^2^	Primary atom site location: structure-invariant direct methods
Least-squares matrix: full	Secondary atom site location: difference Fourier map
*R*[*F*^2^ > 2σ(*F*^2^)] = 0.058	Hydrogen site location: inferred from neighbouring sites
*wR*(*F*^2^) = 0.204	H-atom parameters constrained
*S* = 1.00	*w* = 1/[σ^2^(*F*_o_^2^) + (0.0962*P*)^2^] where *P* = (*F*_o_^2^ + 2*F*_c_^2^)/3
2471 reflections	(Δ/σ)_max_ = 0.013
133 parameters	Δρ_max_ = 1.80 e Å^−^^3^
0 restraints	Δρ_min_ = −0.83 e Å^−^^3^

### Special details

**Table:**

Geometry. All e.s.d.'s (except the e.s.d. in the dihedral angle between two l.s. planes) are estimated using the full covariance matrix. The cell e.s.d.'s are taken into account individually in the estimation of e.s.d.'s in distances, angles and torsion angles; correlations between e.s.d.'s in cell parameters are only used when they are defined by crystal symmetry. An approximate (isotropic) treatment of cell e.s.d.'s is used for estimating e.s.d.'s involving l.s. planes.
Refinement. Refinement of *F*^2^ against ALL reflections. The weighted *R*-factor *wR* and goodness of fit *S* are based on *F*^2^, conventional *R*-factors *R* are based on *F*, with *F* set to zero for negative *F*^2^. The threshold expression of *F*^2^ > σ(*F*^2^) is used only for calculating *R*-factors(gt) *etc*. and is not relevant to the choice of reflections for refinement. *R*-factors based on *F*^2^ are statistically about twice as large as those based on *F*, and *R*- factors based on ALL data will be even larger.

### Fractional atomic coordinates and isotropic or equivalent isotropic displacement parameters (Å^2^)

**Table:**

	*x*	*y*	*z*	*U*_iso_*/*U*_eq_	
I1	0.64794 (15)	0.39539 (8)	0.25072 (11)	0.0679 (5)	
Cu1	0.4723 (2)	0.26167 (12)	0.34694 (15)	0.0456 (6)	
O1	0.5633 (10)	0.2803 (6)	0.5351 (8)	0.044 (2)	
O2	0.3381 (11)	0.2106 (6)	0.1834 (8)	0.038 (2)	
H2O	0.2428	0.2147	0.1437	0.08 (6)*	
N1	0.2813 (13)	0.3302 (7)	0.3748 (9)	0.038 (3)	
N2	0.6101 (12)	0.1521 (7)	0.3455 (9)	0.033 (3)	
C1	0.4697 (16)	0.3364 (10)	0.5848 (13)	0.050 (4)	
H1A	0.5391	0.3755	0.6457	0.06*	
H1B	0.4074	0.3021	0.6276	0.06*	
C2	0.3579 (18)	0.3894 (9)	0.4808 (13)	0.048 (4)	
H2A	0.2761	0.4173	0.5113	0.057*	
H2B	0.4178	0.4349	0.453	0.057*	
C3	0.1879 (19)	0.3814 (10)	0.2666 (13)	0.061 (5)	
H3A	0.0986	0.4086	0.2864	0.092*	
H3B	0.1488	0.3434	0.1954	0.092*	
H3C	0.2551	0.4259	0.247	0.092*	
C4	0.1717 (17)	0.2652 (10)	0.4069 (14)	0.052 (4)	
H4A	0.0854	0.2955	0.427	0.079*	
H4B	0.2304	0.2311	0.4781	0.079*	
H4C	0.1284	0.2271	0.3365	0.079*	
C5	0.4044 (16)	0.1367 (9)	0.1424 (13)	0.046 (4)	
H5A	0.3869	0.1398	0.0523	0.055*	
H5B	0.3528	0.0839	0.1607	0.055*	
C6	0.5814 (15)	0.1335 (9)	0.2087 (11)	0.038 (3)	
H6A	0.6234	0.0759	0.198	0.045*	
H6B	0.637	0.1766	0.1725	0.045*	
C7	0.7829 (17)	0.1630 (11)	0.4054 (13)	0.059 (5)	
H7A	0.8063	0.1534	0.4942	0.089*	
H7B	0.8144	0.2215	0.3905	0.089*	
H7C	0.8413	0.1214	0.3707	0.089*	
C8	0.5534 (19)	0.0790 (9)	0.4089 (13)	0.054 (4)	
H8A	0.5948	0.0246	0.3877	0.08*	
H8B	0.4381	0.0775	0.382	0.08*	
H8C	0.5906	0.0876	0.4982	0.08*	

### Atomic displacement parameters (Å^2^)

**Table:**

	*U*^11^	*U*^22^	*U*^33^	*U*^12^	*U*^13^	*U*^23^
I1	0.0801 (9)	0.0630 (7)	0.0579 (7)	−0.0279 (7)	0.0159 (6)	0.0090 (6)
Cu1	0.0337 (9)	0.0659 (13)	0.0304 (9)	0.0154 (9)	−0.0015 (7)	−0.0125 (9)
O1	0.040 (5)	0.057 (6)	0.032 (5)	0.016 (5)	0.006 (4)	−0.003 (5)
O2	0.030 (6)	0.046 (6)	0.034 (5)	−0.003 (4)	0.004 (4)	−0.012 (5)
N1	0.042 (7)	0.047 (7)	0.021 (6)	0.013 (5)	0.005 (5)	−0.001 (5)
N2	0.033 (6)	0.045 (7)	0.020 (5)	−0.010 (5)	0.005 (5)	−0.001 (5)
C1	0.038 (9)	0.063 (11)	0.046 (9)	0.004 (8)	0.005 (7)	−0.006 (8)
C2	0.050 (9)	0.046 (9)	0.045 (8)	0.003 (8)	0.010 (7)	−0.015 (8)
C3	0.073 (12)	0.062 (11)	0.037 (8)	0.027 (9)	−0.001 (8)	−0.001 (8)
C4	0.048 (9)	0.070 (11)	0.053 (9)	−0.019 (8)	0.035 (8)	−0.008 (8)
C5	0.053 (9)	0.048 (9)	0.032 (8)	0.011 (7)	0.007 (7)	0.005 (7)
C6	0.038 (8)	0.045 (9)	0.030 (7)	0.011 (7)	0.011 (6)	0.014 (6)
C7	0.059 (10)	0.071 (12)	0.042 (9)	0.033 (9)	0.008 (8)	−0.009 (9)
C8	0.081 (12)	0.043 (9)	0.034 (8)	0.000 (8)	0.013 (8)	0.011 (7)

### Geometric parameters (Å, º)

**Table:**

I1—Cu1	2.928 (2)	C2—H2B	0.97
Cu1—O1	2.030 (9)	C3—H3A	0.96
Cu1—N2	2.058 (11)	C3—H3B	0.96
Cu1—N1	2.059 (10)	C3—H3C	0.96
Cu1—O2	2.010 (8)	C4—H4A	0.96
O1—C1	1.399 (16)	C4—H4B	0.96
O2—C5	1.400 (16)	C4—H4C	0.96
O2—H2O	0.82	C5—C6	1.502 (17)
N1—C3	1.466 (15)	C5—H5A	0.97
N1—C2	1.481 (16)	C5—H5B	0.97
N1—C4	1.488 (16)	C6—H6A	0.97
N2—C7	1.465 (16)	C6—H6B	0.97
N2—C8	1.476 (17)	C7—H7A	0.96
N2—C6	1.495 (15)	C7—H7B	0.96
C1—C2	1.512 (19)	C7—H7C	0.96
C1—H1A	0.97	C8—H8A	0.96
C1—H1B	0.97	C8—H8B	0.96
C2—H2A	0.97	C8—H8C	0.96
			
O1—Cu1—N2	93.9 (4)	N1—C3—H3A	109.5
O1—Cu1—N1	82.2 (4)	N1—C3—H3B	109.5
N2—Cu1—N1	155.4 (4)	H3A—C3—H3B	109.5
O1—Cu1—I1	101.0 (3)	N1—C3—H3C	109.5
N2—Cu1—I1	101.3 (3)	H3A—C3—H3C	109.5
N1—Cu1—I1	103.3 (3)	H3B—C3—H3C	109.5
I1—Cu1—O2	99.6 (3)	N1—C4—H4A	109.5
O1—Cu1—O2	159.5 (4)	N1—C4—H4B	109.5
O2—Cu1—N1	92.9 (4)	H4A—C4—H4B	109.5
O2—Cu1—N2	82.3 (4)	N1—C4—H4C	109.5
C1—O1—Cu1	113.3 (7)	H4A—C4—H4C	109.5
C5—O2—H2O	109.5	H4B—C4—H4C	109.5
C3—N1—C2	110.0 (11)	O2—C5—C6	109.0 (11)
C3—N1—C4	108.1 (11)	O2—C5—H5A	109.9
C2—N1—C4	112.7 (11)	C6—C5—H5A	109.9
C3—N1—Cu1	115.2 (9)	O2—C5—H5B	109.9
C2—N1—Cu1	103.5 (8)	C6—C5—H5B	109.9
C4—N1—Cu1	107.4 (8)	H5A—C5—H5B	108.3
C7—N2—C8	108.0 (11)	N2—C6—C5	109.7 (10)
C7—N2—C6	109.3 (10)	N2—C6—H6A	109.7
C8—N2—C6	111.3 (10)	C5—C6—H6A	109.7
C7—N2—Cu1	115.2 (9)	N2—C6—H6B	109.7
C8—N2—Cu1	109.4 (8)	C5—C6—H6B	109.7
C6—N2—Cu1	103.6 (7)	H6A—C6—H6B	108.2
O1—C1—C2	110.1 (11)	N2—C7—H7A	109.5
O1—C1—H1A	109.6	N2—C7—H7B	109.5
C2—C1—H1A	109.6	H7A—C7—H7B	109.5
O1—C1—H1B	109.6	N2—C7—H7C	109.5
C2—C1—H1B	109.6	H7A—C7—H7C	109.5
H1A—C1—H1B	108.2	H7B—C7—H7C	109.5
N1—C2—C1	108.9 (11)	N2—C8—H8A	109.5
N1—C2—H2A	109.9	N2—C8—H8B	109.5
C1—C2—H2A	109.9	H8A—C8—H8B	109.5
N1—C2—H2B	109.9	N2—C8—H8C	109.5
C1—C2—H2B	109.9	H8A—C8—H8C	109.5
H2A—C2—H2B	108.3	H8B—C8—H8C	109.5

### Hydrogen-bond geometry (Å, º)

**Table:**

*D*—H···*A*	*D*—H	H···*A*	*D*···*A*	*D*—H···*A*
O2—H2*O*···O1^i^	0.82	1.68	2.482 (12)	167

Symmetry code: (i) *x*−1/2, −*y*+1/2, *z*−1/2.
